# Neuroendocrine mechanisms underlying estrogen positive feedback and the LH surge

**DOI:** 10.3389/fnins.2022.953252

**Published:** 2022-07-27

**Authors:** Alexander S. Kauffman

**Affiliations:** Department of OBGYN and Reproductive Sciences, University of California, San Diego, La Jolla, CA, United States

**Keywords:** Kiss1, GnRH, RP3V, AVPV, kisspeptin, SCN, ovulation, reproduction

## Abstract

A fundamental principle in reproductive neuroendocrinology is sex steroid feedback: steroid hormones secreted by the gonads circulate back to the brain to regulate the neural circuits governing the reproductive neuroendocrine axis. These regulatory feedback loops ultimately act to modulate gonadotropin-releasing hormone (GnRH) secretion, thereby affecting gonadotropin secretion from the anterior pituitary. In females, rising estradiol (E_2_) during the middle of the menstrual (or estrous) cycle paradoxically “switch” from being inhibitory on GnRH secretion (“negative feedback”) to stimulating GnRH release (“positive feedback”), resulting in a surge in GnRH secretion and a downstream LH surge that triggers ovulation. While upstream neural afferents of GnRH neurons, including kisspeptin neurons in the rostral hypothalamus, are proposed as critical loci of E_2_ feedback action, the underlying mechanisms governing the shift between E_2_ negative and positive feedback are still poorly understood. Indeed, the precise cell targets, neural signaling factors and receptors, hormonal pathways, and molecular mechanisms by which ovarian-derived E_2_ indirectly stimulates GnRH surge secretion remain incompletely known. In many species, there is also a circadian component to the LH surge, restricting its occurrence to specific times of day, but how the circadian clock interacts with endocrine signals to ultimately time LH surge generation also remains a major gap in knowledge. Here, we focus on classic and recent data from rodent models and discuss the consensus knowledge of the neural players, including kisspeptin, the suprachiasmatic nucleus, and glia, as well as endocrine players, including estradiol and progesterone, in the complex regulation and generation of E_2_-induced LH surges in females.

## Introduction

A fundamental tenet of hypothalamic-pituitary-gonadal (HPG) axis regulation is sex steroid feedback: the ability of gonadal steroid hormones (estrogens, androgens, and progestins) to circulate back into the brain and regulate the neural circuits, including gonadotropin-releasing hormone (GnRH) neurons, that govern the HPG neuroendocrine axis. Although feedback loops were proposed decades ago, the detailed mechanisms by which gonadal sex steroids act in the brain to inhibit (“negative feedback”) or stimulate (“positive feedback”) GnRH secretion still remain poorly understood, in part because GnRH cells themselves lack the requisite sex steroid receptors for steroid feedback. Thus, other “upstream” brain cells communicating with GnRH neurons are posited to serve as loci of sex steroid feedback action. Though advances were made in recent years with the discovery of the neuropeptide kisspeptin, the precise brain cells, neural signaling factors and receptors, and physiological and molecular mechanisms by which ovarian-derived estrogen acts in the brain to stimulate GnRH release (“estrogen positive feedback”) still remain major gaps in knowledge. In the present review, we summarize essential background on neuroendocrine mechanisms of estrogen positive feedback, highlight recent advances on this topic, and discuss some critical gaps in knowledge that need addressing to better understand how the LH surge is both generated and modulated. Given other recent in-depth reviews on this and related topics (Herbison, 2008; Christian and Moenter, 2010; Uenoyama et al., 2021; Goodman et al., 2022; Tonsfeldt et al., 2022), we will focus herein on historical and recent data gleaned primarily from rodent models. Readers interested in comparative aspects of estrogen feedback and kisspeptin biology in other species are referred to several other informative reviews (Berga and Naftolin, 2012; Plant, 2012; Lehman et al., 2013; Matsuda et al., 2019; Goodman et al., 2022).

## Estrogen positive feedback and the LH surge

GnRH neurons in the forebrain project fibers to the median eminence to secrete pulsatile GnRH, which activates pituitary secretion of gonadotropin hormones (LH and FSH), in turn driving the synthesis and secretion of gonadal sex steroids [estradiol (E_2_) and testosterone (T)]. Besides regulating reproductive physiology and behavior, circulating E_2_ and T also provide feedback loops to the brain to modulate GnRH secretion. During most of the female cycle, lower levels of ovarian E_2_ provide negative feedback on pulsatile GnRH release, keeping it within a proper homeostatic range (Sarkar and Fink, 1980; Chongthammakun and Terasawa, 1993; Evans et al., 1994; Freeman, 2006; Goodman and Inskeep, 2006; Herbison, 2020). However, rising E_2_ levels at the end of the follicular phase (proestrus in rodents) paradoxically “switch” from being inhibitory to stimulatory, providing positive feedback activation of GnRH cells. This E_2_ positive feedback induces a massive increase in GnRH secretion (the “GnRH surge”; Sarkar et al., 1976; Moenter et al., 1990, 1992; Freeman, 2006; Herbison, 2008, 2020) which causes a large corresponding “LH surge” from the pituitary to trigger ovulation (Freeman, 1994; Goodman and Inskeep, 2006). The mechanisms governing the critical switch between E_2_ negative and positive feedback are still poorly understood.

Importantly, GnRH neurons lack the sex steroid receptors [estrogen receptor α (ERα), androgen receptor, progesterone receptor] that mediate both sex steroid positive and negative feedback (Lubahn et al., 1993; Couse et al., 2003; Wintermantel et al., 2006; Christian et al., 2008; Herbison, 2008; Cheong et al., 2015). Thus, sex steroid control of GnRH secretion is indirect, occurring in other upstream steroid-sensitive brain cells that communicate with GnRH cells. Classic studies identified the medial basal hypothalamus as the key region for sex steroid negative feedback in both sexes, whereas the hypothalamic anteroventral periventricular nucleus (AVPV), located in the preoptic area (POA), was identified as a critical area for E_2_ positive feedback, especially in rodents [reviewed in Herbison (2008), Christian and Moenter (2010), Khan and Kauffman (2012)]. Unlike GnRH cells, the AVPV region contains many ERα-expressing cells (Bloch et al., 1992; Herbison and Theodosis, 1992; Shughrue et al., 1997; Merchenthaler et al., 2004), and many historical studies functionally implicated the AVPV as a key site for E_2_ induction of the LH surge in rodents (Kalra and McCann, 1975; Goodman, 1978; Wiegand et al., 1978, 1980; Wiegand and Terasawa, 1982; Ronnekleiv and Kelly, 1988; Petersen and Barraclough, 1989; Petersen et al., 1989; Le et al., 1997, 1999, 2001; Smith et al., 2005a). More recently, it was determined that virtually all preoptic ERα + afferents to GnRH neurons reside in the AVPV and immediately adjacent rostral periventricular nucleus, a small anatomical continuum now termed the “rostral periventricular nucleus of the 3rd ventricle” (RP3V; Wintermantel et al., 2006; Herbison, 2008).

## Estrogen regulation of kisspeptin neurons

The neuropeptide kisspeptin, encoded by the *Kiss1* gene, directly stimulates GnRH neurons. Humans and rodents lacking either kisspeptin or its receptor, KISS1R, are completely infertile, with low LH and gonadal sex steroids due to diminished GnRH secretion (de Roux et al., 2003; Seminara et al., 2003; d’Anglemont de Tassigny et al., 2007; Lapatto et al., 2007; Topaloglu et al., 2012). KISS1R is expressed in GnRH cells (Irwig et al., 2004; Messager et al., 2005; Semaan and Kauffman, 2015), and kisspeptin potently stimulates GnRH neuron activation and GnRH secretion, thereby causing LH secretion (Gottsch et al., 2004; Irwig et al., 2004; Dhillo et al., 2005; Han et al., 2005; Messager et al., 2005; Navarro et al., 2005; Kauffman et al., 2007c; d’Anglemont de Tassigny et al., 2008). Importantly, pharmacologically blocking kisspeptin signaling inhibits the LH surge (Kinoshita et al., 2005; Pineda et al., 2010; Smith et al., 2011), and E_2_-treated *Kiss1r-* or *Kiss1*-null female mice do not exhibit LH surges or GnRH neuron activation (Clarkson et al., 2008; Dror et al., 2013), indicating that kisspeptin signaling is critical for the LH surge generation.

In all mammals, kisspeptin-synthesizing neurons primarily reside in two distinct hypothalamic areas. In rodents, the largest kisspeptin population is in the arcuate nucleus (ARC) while another more anterior population is in the RP3V (Gottsch et al., 2004; Smith et al., 2005a; Kauffman et al., 2007b; Clarkson et al., 2009; Kauffman, 2010a; Lehman et al., 2013). The RP3V kisspeptin population is one and the same as the originally identified kisspeptin neurons in the AVPV and PeN nuclei (and is therefore often referred to as the AVPV/PeN kisspeptin population). In the present review, we will use the designations RP3V^KISS^ and ARC^KISS^ neurons. Importantly, while both kisspeptin populations stimulate GnRH neurons, RP3V^KISS^ and ARC^KISS^ neurons directly project to different anatomical parts of the GnRH neuron, with the former targeting GnRH soma in the OVLT and POA and the latter targeting GnRH fiber terminals in the MBH and median eminence (Lehman et al., 2013; Yip et al., 2015, 2021). This is no small point, as the anatomical and physical separation of kisspeptin synthesis and release between RP3V^KISS^ and ARC^KISS^ neurons may in fact underlie the ability of estrogen to provide both positive and negative feedback on GnRH neurons by acting on kisspeptin neurons in different brain locations for each process (Wang et al., 2020). Indeed, and to this point, *Kiss1* gene expression is strongly regulated by gonadal sex steroids (E_2_ and T) in a region-specific manner: for both sexes, E_2_ or T *increases Kiss1* mRNA levels in the RP3V whereas these sex steroids reduce *Kiss1* mRNA levels in the ARC (Smith et al., 2005a,b; Kauffman et al., 2007b). Conversely, when circulating sex steroids are low or absent [e.g., diestrus or ovariectomized (OVX) females], *Kiss1* mRNA levels decrease in the RP3V and increase in the ARC (Smith et al., 2005a,b; Kauffman et al., 2007b,^2009^). Both RP3V^KISS^ and ARC^KISS^ neurons express high levels of ERα (Smith et al., 2005a; Adachi et al., 2007; Poling et al., 2017), and we and others, including seminal experiments from the Steiner lab (Smith et al., 2005a), have shown that E_2_’s effects on both RP3V and ARC *Kiss1* mRNA levels are direct and occur specifically *via* ERα signaling (Smith et al., 2006; Gottsch et al., 2009; Dubois et al., 2015, 2016; Stephens et al., 2016; Poling et al., 2017; Stephens and Kauffman, 2021).

The seemingly simple finding that E_2_ regulates the *Kiss1* gene differently in the two hypothalamic *Kiss1*-expressing populations has ultimately proven to be a crucial discovery in understanding sex steroid feedback loops. Indeed, this finding led to the proposal that differential effects of E_2_ on *Kiss1* mRNA in the ARC and RP3V reflect different roles of the two kisspeptin populations (Smith et al., 2005a), with ARC^KISS^ neurons participating in E_2_ negative feedback and RP3V^KISS^ neurons participating in E_2_ positive feedback. The proposed role of ARC^KISS^ neurons in negative feedback control of GnRH pulse secretion has been reviewed in detail elsewhere (Herbison, 2018; Moore et al., 2018; Goodman et al., 2022) and will not be discussed here other than by simply summarizing that sex steroids are known to exert negative feedback regulation indirectly on GnRH pulses by acting in the ARC region (Ferin et al., 1974; Smith and Davidson, 1974; Scott et al., 1997), and direct inhibition of ARC^KISS^ neurons, which comprise the “GnRH pulse generator” (Han et al., 2015, 2019; Qiu et al., 2016; Clarkson et al., 2017; McQuillan et al., 2019), may be how E_2_ and T regulate the frequency and amplitude of GnRH (and LH) pulses.

**FIGURE 1:**
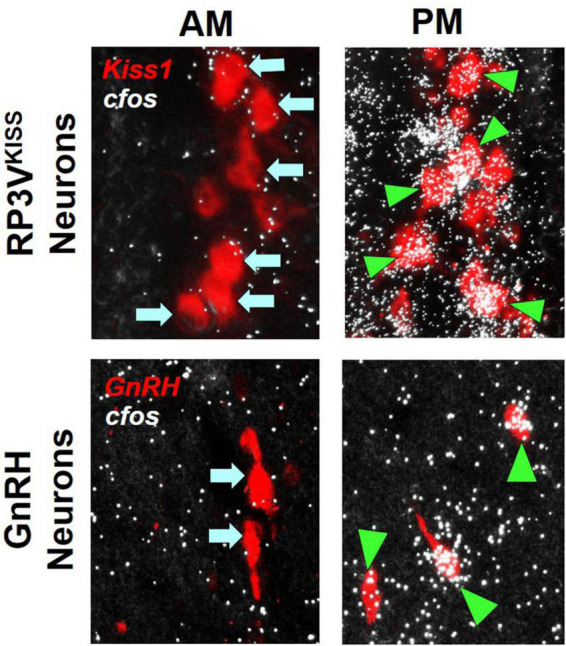
Examples of RP3V^KISS^ and GnRH neuron activation, as indicated by robust *cfos* mRNA co-expression (white “silver grains”; assayed with radiolabeled *in situ* hybridization), during an E_2_-induced LH surge in female mice. The surge typically occurs in the late afternoon/early evening (PM) but not in the morning (AM), matching the higher degree of *cfos* mRNA induction in both RP3V^KISS^ and GnRH neurons in the PM than AM. Green triangles denote example “activated” RP3V^KISS^ or GnRH cells co-expressing *cfos;* blue arrows denote example non-activated cells lacking *cfos*. Adapted from Poling et al. (2017).

**FIGURE 2:**
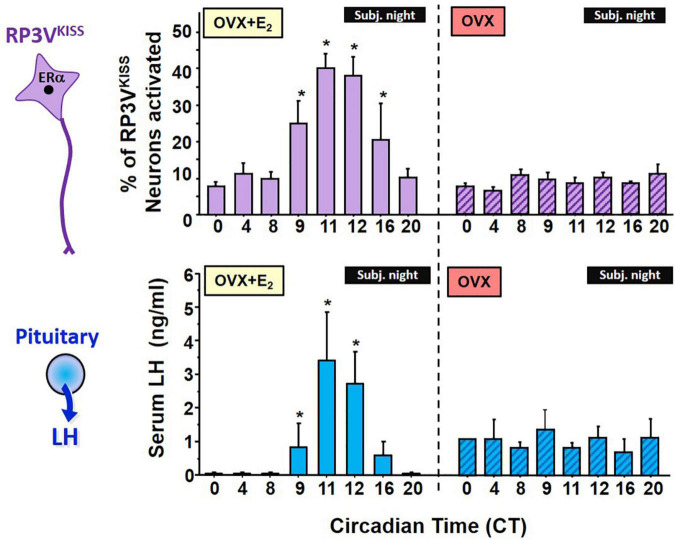
Circadian changes in RP3V^KISS^ neuron activation, measured by *cfos* mRNA induction, and serum LH levels in OVX + E_2_ female mice housed in constant darkness (DD). The onset and peak of RP3V^KISS^ neuron activation mirrors the rise and peak of LH surges, which occur right before the onset of subjective night (defined as the daily onset of locomotor activity, a robust circadian behavioral measure). For comparison, a separate cohort of OVX females not receiving E_2_ were similarly studied but showed no circadian increases in either RP3V^KISS^ neuron activation or serum LH levels. Note that baseline LH levels in the OVX females are higher than in OVX + E_2_ due to lack of gonadal steroid negative feedback in the former group. *, Significantly different than baseline levels at CT 0. Adapted from Robertson et al. (2009).

Contrasting the role of ARC^KISS^ neurons in driving GnRH pulses and mediating steroid feedback, it is now widely believed that RP3V^KISS^ neurons comprise the neural conduit mediating E_2_ positive feedback on GnRH neurons in female rodents (thereby triggering the GnRH and LH surges and subsequent ovulation; Kauffman et al., 2007a; Herbison, 2008). As noted above, RP3V^KISS^ cells express abundant ERα and strongly increase *Kiss1* gene expression in response to elevated E_2_. We and others have also shown that RP3V^KISS^ neurons display increased neuronal activation (*cfos* mRNA or Fos protein) at the time of the LH surge in proestrus or OVX + E_2_-treated females (Figures 1, 2), but not in diestrus or OVX females with insufficient E_2_ (Smith et al., 2006; Adachi et al., 2007; Clarkson et al., 2008; Robertson et al., 2009; Figure 2). Moreover, female mice lacking ERα in kisspeptin neurons do not generate LH surges in response to E_2_ (Dubois et al., 2015); however, because ERα was deleted from all kisspeptin cells, that study was not able to pinpoint the effect specifically to RP3V^KISS^ neurons. However, another study implementing AAV-mediated partial knockdown of ERα in RP3V^KISS^ cells lowered the LH surge magnitude in proestrus and OVX + E_2_ mice (Wang et al., 2019). Finally, as discussed further below, the LH surge is gated by a circadian clock (de la Iglesia and Schwartz, 2006), and RP3V^KISS^ neurons display a circadian pattern of neuronal activation in perfect synchrony with the timing of the LH surge (Robertson et al., 2009).

Additional electrophysiological evidence that supports that RP3V^KISS^ neurons are positively regulated by estrogen, including reported stimulatory effects on ion currents in these cells in mice (Piet et al., 2013; Zhang et al., 2015; Wang et al., 2016; Starrett et al., 2021). Collectively, such studies conclude that normal estrous cycle-driven rises in circulating E_2_ induce increases in overall action potential generation and burst firing in RP3V^KISS^ neurons on proestrus (when E_2_ is elevated) by regulating multiple intrinsic currents in these kisspeptin cells (Wang et al., 2016). Moreover, evidence that increased electrical activity in these cells is sufficient to drive kisspeptin release and, consequently, electrical activity in GnRH neurons and downstream LH secretion provides further support for RP3V^KISS^ neurons mediating and conveying E_2_ positive feedback information to GnRH neurons. Indeed, either continuous or bursting mode optogenetic activation of RP3V^KISS^ neurons in female mouse brain slices reliably generated long-lasting activation of GnRH neuron firing, and optogenetic activation of RP3V^KISS^ neurons *in vivo* generated large increases in LH secretion resembling the endogenous LH surge (Piet et al., 2018). This was supported by a similar report in mice that high-frequency photostimulation of RP3V^KISS^ neurons evokes increased electrical activity in GnRH neurons in several *in vitro* slice orientations (Qiu et al., 2016). Importantly, although RP3V^KISS^ neurons co-express many additional releasable signaling factors along with kisspeptin itself (Stephens and Kauffman, 2021), similar *in vivo* optogenetic activation of RP3V^KISS^ neurons in which kisspeptin had been deleted did not induce LH secretion (Piet et al., 2018), indicating that kisspeptin peptide is a required signaling factor being released from RP3V^KISS^ neurons to induces surges. This does not rule out important involvement of other co-released peptides or neurotransmitters, but clearly designates kisspeptin as one essential player for the surge process.

In rodents, E_2_’s positive effects on both LH surge generation and RP3V^KISS^ cells are sexually dimorphic: E_2_-treated male rodents have lower RP3V *Kiss1* mRNA levels than E_2_-treated females (Kauffman et al., 2007b; Poling et al., 2017), and male RP3V^KISS^ neurons are not activated by elevated levels E_2_ (Poling et al., 2017), correlating with male rodents’ inability to generate E_2_-induced LH surges (Buhl et al., 1978; Homma et al., 2009; Poling et al., 2017). Interestingly, developmental manipulations that reverse the sexual differentiation of RP3V^KISS^ neurons (Kauffman et al., 2007b; Homma et al., 2009) similarly reverse the ability to generate LH surges, such that males with female-like RP3V^KISS^ neurons can produce an LH surge whereas females with male-like RP3V^KISS^ neurons can no longer generate LH surges (Homma et al., 2009). Overall, these data, along with the findings in the preceding paragraph, suggest that RP3V^KISS^ neurons participate in generating the sex-specific LH surge during E_2_ positive feedback. Conversely, as noted further below, rodent ARC^KISS^ cells are strongly inhibited by E_2_ and are not highly activated during the LH surge (Smith et al., 2005a; Adachi et al., 2007) [though both ARC^KISS^ and POA kisspeptin neurons may participate in the LH surge in some species (Smith, 2009; Smith et al., 2010; Watanabe et al., 2014; Vargas Trujillo et al., 2017)].

## Circadian regulation of the LH surge and RP3V^KISS^ neurons

The E_2_-induced LH surge is under temporal control (Alleva et al., 1971; Norman and Spies, 1974; Legan and Karsch, 1975; Seibel et al., 1982; Cahill et al., 1998; Kerdelhue et al., 2002; Mahoney et al., 2004; de la Iglesia and Schwartz, 2006). In female rodents, the LH surge is timed to occur exclusively in the late afternoon/early evening of proestrus, thereby aligning subsequent ovulation and female mating and ensuring reproductive success (Kauffman, 2010b). In mammals, the primary circadian clock located in the hypothalamic suprachiasmatic nucleus (SCN) governs circadian rhythms of many biological processes, from gene expression to physiology and behavior. Evidence in rodents indicates the circadian clock in the SCN governs the LH surge timing. First, historical studies showed that not only does the LH surge consistently and predictably occur at a specific time of day, but barbiturate treatment delays the occurrence of the LH surge exactly 24 h until the late afternoon of the next day, suggesting an internal time-keeping component for the surge event (Everett et al., 1949; Sawyer et al., 1949; Everett and Sawyer, 1950; Siegel et al., 1976; Stetson and Watson-Whitmyre, 1977). Second, OVX females do not surge, emphasizing the requisite role for E_2_ in surge induction, but OVX females given elevated E_2_ (OVX + E_2_) display an LH surge which occurs solely around the time of lights off and which repeats daily at the same time as long as E_2_ is elevated (Caligaris et al., 1971; Norman et al., 1973; Norman and Spies, 1974; Legan and Karsch, 1975; Legan et al., 1975). Third, experimental phase shifts or genetic alterations of behavioral circadian rhythms known to be timed by the SCN (e.g., circadian locomotor activity) phase shift the timing of the LH surge, with the onset and timing of the new surge always coupled to the new timing of locomotor activity onset (Alleva et al., 1971; Fitzgerald and Zucker, 1976; Moline and Albers, 1988; Lucas et al., 1999; Smarr et al., 2012). Fourth, experimentally induced “splitting” of the lateral SCN hemispheres of hamsters by exposure to constant light elicits 2 daily LH surges ∼12 h apart, along with lateralized GnRH neuron activation (Swann and Turek, 1985; de la Iglesia et al., 2003). These two daily “split” LH surges are thought to be separately caused by activation of left and right hemisphere GnRH neuron populations alternatingly activated 12 h apart by the two sides of the SCN. Further supporting this possibility, SCN neurons are activated right before the onset of the LH surge, as measured by increased Fos expression (Tsukahara, 2006). Lastly, in female rats and hamsters, physical destruction of the SCN (Bishop et al., 1972; Brown-Grant and Raisman, 1977; Gray et al., 1978; Kawakami et al., 1980; Wiegand et al., 1980; Wiegand and Terasawa, 1982; Ronnekleiv and Kelly, 1986, 1988; Palm et al., 1999), prevents LH surges, even in the presence of elevated E_2_, a finding supported by similar observations of impaired LH surges and reproductive dysfunction in clock gene knock out mice (Miller et al., 2004; Boden et al., 2010; Chu et al., 2013).

**FIGURE 3:**
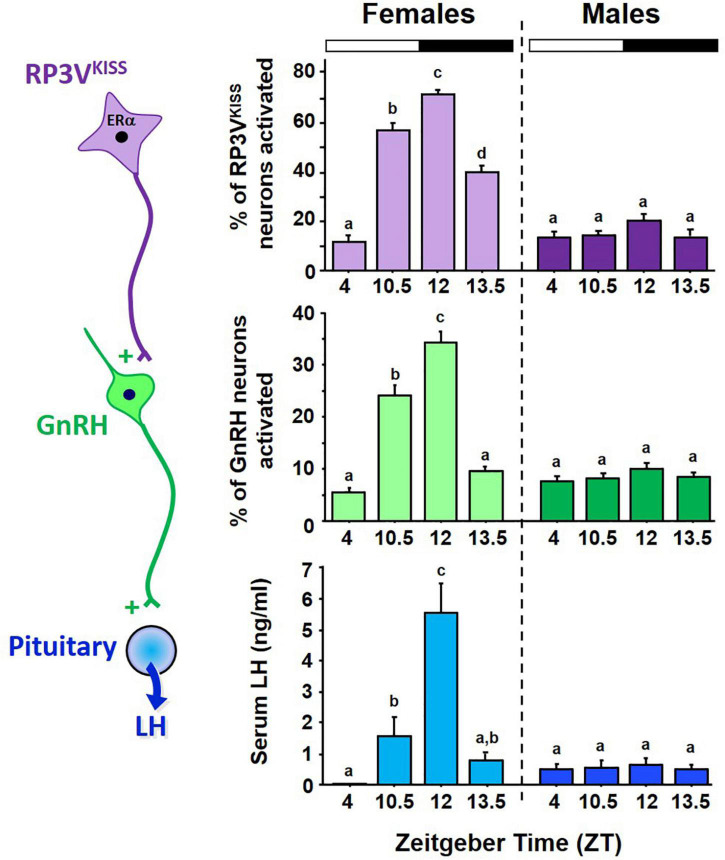
Circadian changes in RP3V^KISS^ and GnRH neuron activation, measured by *cfos* induction using double ISH, and serum LH in female mice housed in 12:12 LD cycle. All females were OVX + E_2_. The onset and peak of neuron activation occurs in synchrony with the rise and peak of LH levels indicative of LH surges. For comparison, male littermates were housed in same 12:12 conditions and similarly GDX + E_2_, yet showed no circadian increases in RP3V^KISS^ neuron activation, GnRH neuron activation, or serum LH levels. Bars with different letters above them are significantly different from each other. Adapted from Poling et al. (2017).

If RP3V^KISS^ neurons participate in the LH surge process, do these neurons demonstrate a circadian component that may relate to the timing of the LH surge? Anatomically, the RP3V region receives SCN axonal projections (de la Iglesia et al., 1995; Watson et al., 1995), suggesting there could be SCN-derived circadian input on neurons there. Our lab therefore first tested whether RP3V^KISS^ neurons of female mice exhibit circadian changes and if such changes occur in synchrony with the timing of the LH surge (Robertson et al., 2009). We found that OVX + E_2_ mice housed in constant darkness (to remove light cues and allow their circadian clock to free-run on its endogenous period) showed circadian increases in both R3PV *Kiss1* mRNA levels and RP3V^KISS^ neuron activation (Figure 2). Moreover, these temporal changes in RP3V^KISS^ measures peaked just before the onset of subjective night [defined as the onset of daily locomotor activity; circadian time (CT) 12], with lower levels earlier in the subjective morning or later in the subjective night. Importantly, in these females, the observed circadian increases in RP3V *Kiss1* levels and RP3V^KISS^ neuron activation occurred synchronously with the circadian onset and duration of the LH surge (Robertson et al., 2009; Figure 2). These data were later confirmed by us and others in subsequent studies of female rodents housed in light-dark cycles (Figure 3) or in SCN lesioned females (Williams et al., 2011; Smarr et al., 2012, 2013; Poling et al., 2017), supporting the notion that circadian activation of RP3V^KISS^ may underlie the circadian nature of the LH surge.

Interestingly, we also demonstrated that adult OVX mice without E_2_ supplementation lack any circadian changes in LH secretion, RP3V *Kiss1* levels, or RP3V^KISS^ neuron activation, with all measures being the same at all time points (Robertson et al., 2009; Figure 2). Thus, the circadian regulation of RP3V^KISS^ is dependent on elevated E_2_, supported by similar data of dampened circadian increases in RP3V kisspeptin neuron activation in OVX female hamsters or in *Kiss1* mRNA levels in diestrus versus proestrus mice (Williams et al., 2011; Chassard et al., 2015). These findings may explain, in part, why OVX (no E_2_) and diestrus females (low E_2_) do not display an LH surge, though the underlying reason for the E_2_-dependence of RP3V kisspeptin neuron circadian changes is still not entirely known. As noted below, the vasopressin receptor, V1a, is E_2_-sensitive (Kalamatianos et al., 2004), and this might contribute, in part, to the lack of RP3V^KISS^ neuron activation in OVX and diestrus females. Indeed, vasopressin treatment stimulates RP3V^KISS^ neuron activity in OVX + E_2_ but not OVX mice (Piet et al., 2015), indicating E_2_ may enhance the sensitivity of RP3V^KISS^ neuron to vasopressin. In some cases, E_2_ also has also been shown to modulate synaptic transmission to RP3V^KISS^ neurons, increasing stimulatory glutamate transmission and decreasing inhibitory GABAergic transmission to these neurons (DeFazio et al., 2014; Wang et al., 2018), though this has not always been consistently observed.

Because of sexual differentiation, the brains of male rodents, unlike their female counterparts, are incapable of generating LH surges in response to elevated E_2_. We recently showed that adult E_2_-treated male mice also demonstrate no circadian changes in GnRH cell activation, *Kiss1* mRNA, or RP3V^KISS^ neuron activation (Poling et al., 2017; Figure 3). Thus, in addition to synthesizing less kisspeptin in the RP3V than females (Clarkson and Herbison, 2006; Kauffman et al., 2007b; Poling et al., 2017), males’ RP3V^KISS^ neurons also do not become activated by E_2_ at any point of the circadian cycle, indicating additional sexual dimorphisms beyond just *Kiss1* gene expression. Interestingly, we showed that male mice have lower levels of ERα co-expression in RP3V^KISS^ neurons than do females (Poling et al., 2017), though male RP3V^KISS^ neurons still had a decent degree of ERα and it remains unknown if the ERα sex difference explains the complete lack of neuron activation in males. Unraveling exactly why E_2_-treated males lack any circadian RP3V^KISS^ changes, or why the circadian activation of RP3V^KISS^ neuron is E_2_-dependent in females, could provide valuable mechanistic insight for how E_2_ positive feedback normally operates.

## Possible suprachiasmatic nucleus circuits regulating gonadotropin-releasing hormone and RP3V^KISS^ neurons

The SCN is anatomically and functionally divided into the ventrolateral “core” and the dorsomedial “shell.” The ventrolateral neurons receive direct light input from the retinohypothalamic tract and relay this photic information to other SCN neurons, including dorsomedial neurons (Abrahamson and Moore, 2001). Each SCN neuron is rhythmic on its own (Welsh et al., 1995) but the nucleus as a whole synchronizes among itself to produces a single robust rhythmic output that can govern circadian changes in many other brain areas (Abrahamson and Moore, 2001; Deurveilher and Semba, 2005; Welsh et al., 2010). Different SCN neurons produce a variety of neuropeptides, including vasoactive intestinal polypeptide (VIP), arginine vasopressin (AVP), gastrin-releasing peptide, prokineticin 2, neuromedin S, and substance P, as well as GABA (Cheng et al., 2002; Antle and Silver, 2005; Lee et al., 2015; Shan et al., 2020; Wen et al., 2020). VIP and AVP are synthesized in the ventrolateral and dorsomedial SCN, respectively, (Card et al., 1988). Both *Vip* mRNA levels and VIP release demonstrate *in vitro* circadian rhythmicity in SCN slices (Shinohara et al., 1999, 2000; Dardente et al., 2004). Findings from transgenic mice lacking either VIP or VIPR2 (aka VPAC_2_, a VIP receptor) suggest that VIP is an important communicator to extra-SCN brain areas because these knockout mice display disrupted or altered circadian rhythms, and sometimes arrhythmicity (Harmar et al., 2002; Colwell et al., 2003). Likewise, AVP also demonstrates circadian rhythmicity, with highest levels during the subjective day (Reppert et al., 1981). Although AVP-deficient Brattleboro rats show intact circadian rhythmicity for several behaviors (Peterson et al., 1980; Groblewski et al., 1981) other studies suggest that AVP is an important SCN output signal for some physiological circadian rhythms, including daily stress hormone secretion (Kalsbeek et al., 1992, 1996a,1996b) and possibly reproductive hormone secretion (discussed below), and SCN AVP neurons project to several hypothalamic regions, including the PVN, DMN, POA, and RP3V (Dai et al., 1998; Kalsbeek et al., 2008).

**FIGURE 4:**
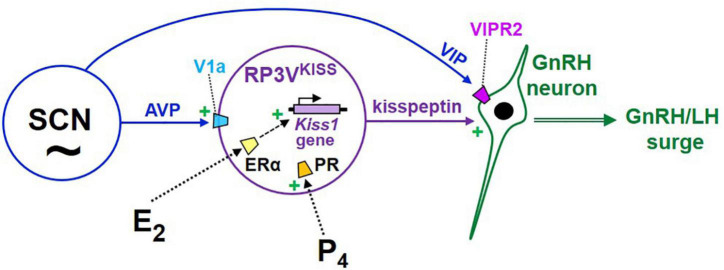
Cartoon model of the proposed hypothalamic neural circuits regulating the E_2_-induced preovulatory LH surge in female rodents. Under this model, GnRH neurons are activated by upstream kisspeptin neurons in the RP3V, which are E_2_-sensitive and express both ER and PR. RP3V^KISS^ neurons are themselves also influenced by both progesterone and circadian cues, the latter likely arising from the SCN, including vasopressin neurons. In addition, VIP neurons in the SCN project to GnRH neurons and may be involved in additional modulation of GnRH secretion during E_2_ positive feedback.

How might the SCN clock communicate circadian timing information to the GnRH system to help time the occurrence of the LH surge? At least two neural anatomical pathways (1 direct, 1 indirect) may link the SCN to GnRH neurons (Figure 4). A proposed direct pathway involves VIP neurons in the ventrolateral SCN that directly target GnRH neurons (van der Beek et al., 1993; Van der Beek et al., 1997), which express the VIP receptor VIPR2 (Van der Beek et al., 1997; Smith et al., 2000; Figure 4). Early data in female rats indicated that many GnRH neurons that express Fos at the time of the LH surge also receive VIP-immunoreactive contacts (Lee et al., 1990a; van der Beek et al., 1994; Harney et al., 1996), suggesting that GnRH-surge generating neurons may be under regulation by VIP. However, on its own this remains a correlational line of evidence that does not demonstrate a functional role for VIP in activating GnRH neurons at the time of the surge, and also does not consider the likelihood that Fos-expressing GnRH neurons may also be targeted by other non-VIP neurons; that is, whether the observed Fos induction in GnRH is actually caused by SCN^VIP^ neurons or VIP peptide signaling still requires direct demonstration. Still, the observation in rats that SCN VIP neurons anatomically project to GnRH neurons is often cited as evidence that this circuit likely plays a role in the GnRH surge.

At present, there is only limited, and mostly indirect, functional evidence that the SCN^VIP^→GnRH circuit is important for the LH surge: (1) *in vitro* VIP treatment can directly stimulate GnRH neuron electrical activity in female mouse brain slices (Christian and Moenter, 2008; Piet et al., 2016); and (2) in rats, antisense lowering of *Vip* mRNA levels in the SCN delays and reduces (but does not abolish) the *in vivo* LH surge and lowers GnRH neuron activation (Harney et al., 1996; Gerhold et al., 2005). This was echoed by another study in rats showing that centrally administered VIP antiserum blunts the amplitude of the LH surge and delays its onset (van der Beek et al., 1999), though the exact neuroanatomical site of action could not be determined in that study. Contrastingly, another study reported that central (icv) antibody blockade of VIP signaling did not alter the LH surge (Murai et al., 1989), though this may be confounded by lack of specificity in the regional and cell targets of central icv infusions. Promisingly, female *Vip* KO mice have circadian disruptions and are subfertile, including ovulatory deficits (Colwell et al., 2003; Loh et al., 2014), though the presence or timing of the LH surge has not been directly studied and VIP is absent from all brain areas in these KOs, limiting interpretation. Like the antisense *Vip* knockdown study, the *Vip* KO findings do not tease apart a possible direct effect of SCN-derived VIP output on GnRH neurons versus an indirect effect of disabled VIP signaling elsewhere or locally within the SCN (important for daily clock function). Still, these limited data suggest VIP may facilitate some aspect of GnRH surge timing, though more compelling direct evidence is still needed, especially *in vivo*. Indeed, while VIP activates GnRH neuron electrical activity *in vitro*, several studies report that central VIP injections in young adult female rats surprisingly inhibit the LH surge and GnRH neurons (Weick and Stobie, 1992; Harney et al., 1996; Kauffman et al., 2014), *via* mechanisms currently unknown. Again, a caveat is that icv VIP treatment may act on multiple brain areas and cell types, including but not limited to RP3V^KISS^ cells, which themselves were also inhibited by icv VIP injection (Kauffman et al., 2014), complicating interpretation. Which VIP receptor that might be mediating effects is also not determined; RP3V^KISS^ and GnRH neurons do not readily express VPIR1, only VIPR2 (Burger et al., 2018; Stephens and Kauffman, 2021), making this receptor a leading candidate. However, other neurons involved may express VPIR1 and its involvement cannot be ruled out. Moreover, electrophysiology recent evidence suggests that VIP may directly communicate with RP3V^KISS^ cells in an excitatory manner during diestrus (Mansano et al., 2022); what the relevance of such diestrus VIP signaling is for the LH surge process and whether this also occurs on proestrus is not yet known.

An indirect SCN→GnRH circuit has been proposed with AVP neurons in the dorsomedial SCN targeting and stimulating RP3V^KISS^ neurons (Figure 4), which then activate GnRH neurons (de la Iglesia et al., 1995; Watson et al., 1995; Leak and Moore, 2001; Kriegsfeld et al., 2004; Herbison, 2008). Anatomically, some SCN*^AVP^* neurons project to the RP3V region, including RP3V^KISS^ neurons (Williams et al., 2011; Jamieson et al., 2021) which express V1a (AVP receptor; Williams et al., 2011; Stephens and Kauffman, 2021; Figure 4). This finding of AVP-ir fiber targeting of RP3V^KISS^ neurons has been confirmed at the electron microscopy level in female mice (Vida et al., 2010). Importantly, lesioning the SCN in hamsters removed most AVP appositions on RP3V^KISS^ neurons, suggesting that the primary source of AVP input is the SCN, at least in this species (Williams et al., 2011). In female mice, more kisspeptin neurons show AVP appositions with E_2_ treatment (Vida et al., 2010); by contrast, in the same animals, VIP connections to RP3V^KISS^ neurons were reported to be far less prevalent, regardless of E_2_ treatment (Vida et al., 2010). Supporting this anatomical data are correlational findings that SCN AVP levels are circadian, peaking in the late subjective day similar to circadian increases in RP3V^KISS^ neurons and the LH surge (Reppert et al., 1981; Krajnak et al., 1998), and E_2_ increases *V1a* levels in the POA area (which contains the RP3V; Funabashi et al., 2000; Kalamatianos et al., 2004).

Functionally, *in vitro* AVP treatment or optogenetic stimulation of SCN*^AVP^* neural fibers in POA brain slices is sufficient to stimulate *in vitro* RP3V^KISS^ electrical activity *via* V1a receptor (Piet et al., 2015; Jamieson et al., 2021). This is supported by limited *in vivo* data that icv AVP injection increases LH secretion in arrhythmic *Clock* KO mice that normally lack LH surges (though RP3V^KISS^ was not studied; Miller et al., 2006) and stimulates Fos in hamster RP3V^KISS^ neurons (though LH was not measured; Williams et al., 2011). Although those 2 studies did not determine where in the brain the icv AVP injections acted, AVP infused directly into the POA induced an LH surge in SCN-lesioned OVX + E_2_ rats (Palm et al., 1999). In all cases, it was not determined if the *in vivo* AVP effects were due to direct or indirect action on RP3V^KISS^ neurons (indeed, RP3V^KISS^ was not examined in 2 of the 3 studies). However, another study in SCN-lesioned OVX + E_2_ rats (that do not normally show LH surges) used reverse microdialysis to increase extracellular AVP levels specifically in the POA/RP3V area which then led to LH surge-like secretion (Palm et al., 1999). Interestingly, exogenous AVP treatment is effective at inducing the LH surge in the late afternoon, but not at other times of the day, but the reason for this has not yet been determined (Palm et al., 2001).

Promising *in vivo* evidence showed that central V1a antagonist infusion blocks the occurrence of normal circadian-timed LH surges in female rats on proestrus (Funabashi et al., 1999), implicating endogenous AVP in the LH surge process, though again the target site(s) of action of such central infusion was not determined, nor was the possible neuroanatomical source of AVP (which is also made in non-SCN cells and other brain areas). Moreover, pharmacological blockade of V1a receptors did not result in blunting of the surge in another rat study (Palm et al., 2001), leaving the issue unresolved. Indeed, a third study reported that while a V1a antagonist could prevent stimulatory effects of AVP infusion on LH secretion in Clock mutant mice, suggesting this surge-like secretion is mediated by V1a, the V1a antagonist failed to prevent endogenous proestrous LH surges in WT mice (Miller et al., 2006). That finding suggests that either exogenous AVP treatments are not triggering the actual LH surge generating circuitry or that AVP is *sufficient* for triggering a surge but not *necessary*; the latter possibility could be true if additional pathways or factors are also sufficient to activate the endogenous surge in the absence of AVP action. Clearly this is an important and complex issue that needs further addressing. Unfortunately, AVP KO mice cannot be studied for LH surges because they die in development (Zelena, 2017), but female Brattleboro rats (with a spontaneous AVP gene mutation) are subfertile, including reduced conception rates and small litters (Boer et al., 1981, 1982), perhaps due to impaired LH surges (not yet studied).

The collective findings above suggest AVP may induce LH secretion perhaps by activating RP3V^KISS^ cells, though direct regulation of RP3V^KISS^ neurons *in vivo* has not yet been determined, and “downstream” GnRH neuron activation was often not also studied. Finally, while direct SCN^VIP^ connections to RP3V^KISS^ neurons are reportedly uncommon (Vida et al., 2010; Williams et al., 2011), the possibility of other non-AVP SCN direct projections (or other co-released factors from AVP neurons) to RP3V^KISS^ has not been well studied. Similarly, the SCN projects to other target brain regions besides GnRH neurons and RP3V^KISS^, which could possibly permit additional indirect SCN anatomical pathways to participate in the gating of the GnRH surge. Indeed, a recent study suggested that GABA signaling arising from the SCN may play a regulatory role in preventing RP3V^KISS^ neuron activation at non-surge times (Jamieson et al., 2021); however, such GABA effects were proposed to be indirect on RP3V^KISS^ neurons and mediated *via* a multi-synaptic pathway that involves one (or more) intermediary neurons. In addition to possible polysynaptic effects, extra-synaptic mechanisms are also possible, but this needs to be tested. While intriguing, this hypothesis awaits confirmatory findings and additional studies are needed to identify the location and phenotype of any possible intermediary neurons. In addition, several studies previously suggested that SCN-regulated RFRP-3 neurons in the DMN may provide inhibitory regulation on LH surge generation by acting on GnRH or kisspeptin neurons (Kriegsfeld, 2006; Anderson et al., 2009; Khan and Kauffman, 2012; Rizwan et al., 2012; Poling et al., 2013); it was demonstrated that RFRP-3 neuron activation is reduced in females in the early evening, coincident with the LH surge (Gibson et al., 2008; Poling et al., 2017), suggesting that a reduction in inhibitory signaling by RFRP-3 directly or indirectly to GnRH neurons may be a component to the LH surge process. While intriguing, a similar circadian decline in RFRP-3 also occurs in males (Poling et al., 2017), despite their lack of a surge, suggesting this temporal change may be unrelated to the surge event. Moreover, chronically activating RFRP-3 neurons in transgenic female mice to, in theory, provide long-lasting inhibitory input to the reproductive axis does not impact normal fertility or litters (Mamgain et al., 2021); it may be that RFRP-3 serves as a modulator that can blunt the surge under inhibitory physiological conditions, such as during stress or metabolic challenge, rather than being a requisite component of the normal surge process.

## Possible circadian components intrinsic to RP3V^KISS^ and gonadotropin-releasing hormone neurons

The circadian nature of the GnRH and LH surge, the demonstrated circadian pattern of RP3V^KISS^ activation, and the abolition of the surge in SCN-lesioned females have suggested a role for the SCN in timing surge generation. However, such an involvement of the SCN does not preclude an important contribution of endogenous molecular circadian clocks in other neural populations. Molecular clocks have been demonstrated in many non-SCN cell types, leading investigators to study whether clock genes in kisspeptin neurons may also be expressed and promote intrinsic circadian rhythms. Although temporal changes in daily clock gene expression of *Per1* and *Bmal* (also known as *Arntl*) mRNA were reported in the RP3V region of adult female rats (Smarr et al., 2013), that study did not examine clock gene expression specifically in kisspeptin neurons. As such, it was not possible to conclude that the observed changes were occurring in kisspeptin neurons and/or other RP3V cell types. A subsequent study in female mice examined co-expression of PER1, a core clock protein, in RP3V^KISS^ neurons to determine if these neurons contain a circadian oscillator that helps time downstream activation of GnRH secretion (Chassard et al., 2015). Most RP3V^KISS^ neurons were shown to express PER1 with an E2-sensitive daily rhythm (Chassard et al., 2015). However, whether this observed PER1 rhythm is functionally relevant for the surge process was not determined. Moreover, as pointed out by the authors, the presence of rhythmic PER1 in kisspeptin cells is not sufficient on its own to prove the existence of a circadian oscillator in these cells, as the observed PER1 rhythm in kisspeptin neurons could in theory be driven by upstream SCN input (Chassard et al., 2015). The same study analyzed *in vitro* Per2-luciferase expression in RP3V brain slices (lacking the SCN) and demonstrated a circadian rhythm, though a limitation was that the Per2 expression in this case was not specific to kisspeptin neurons and could be in any number of the many heterogeneous cells in the RP3V region. Still, it is important evidence that circadian rhythms can persist in the RP3V without the input of the SCN; future studies can ascertain if this SCN-independent rhythm occurs specifically in kisspeptin cells.

At present, no study has yet assessed clock gene expression patterns in kisspeptin neurons in the absence of a functional SCN. However, two recent studies approached this issue from another angle by selectively deleting the *Bmal* gene from just kisspeptin neurons using Cre/lox technology. Both studies found that conditional loss of *Bmal* in kisspeptin neurons does not impact fertility (Bittman, 2019; Tonsfeldt et al., 2019). One study reported unaltered LH surges at the normal circadian time (Tonsfeldt et al., 2019) while the other study observed the lack of consistent LH surges over a 5-h period (Bittman, 2019). The reason for the discrepancy between studies is unknown but regardless, overall reproductive success was unaltered, unlike global *Bmal* KO mice which are infertile. Thus, while RP3V^KISS^ neurons clearly exhibit circadian patterns of *Kiss1* gene expression and neuronal activation and receive anatomical input from the SCN, the possibility that RP3V^KISS^ neurons are themselves autonomous circadian clocks still requires more compelling supporting evidence. Interestingly, global *Bmal* KO female mice reportedly can ovulate, though at a reduced rate (Boden et al., 2010); however, their LH surges appear to be absent (Chu et al., 2013), so the endocrine mechanism driving some ovulatory events in these KO mice is unknown and needs further addressing.

Limited evidence suggests that kisspeptin’s stimulation of GnRH cells may also be circadian-gated (Williams et al., 2011). In female hamsters, AVP treatment was shown to increase Fos expression in RP3V^KISS^ neurons in both the morning and afternoon, but surprisingly, similar AVP treatment did not increase Fos in GnRH neurons (Williams et al., 2011). Specifically, GnRH neurons were not activated by AVP treatment in the morning and were also irresponsive in the afternoon, perhaps because they were already activated, although this remains to be studied further. The authors proposed that GnRH neurons possess an intrinsic gating mechanism that modulates their circadian responsiveness to kisspeptin input, thereby making GnRH neurons more sensitive to kisspeptin in the afternoon than the morning. Circadian differences in the ability of kisspeptin treatments to evoke LH secretion *in vivo* has not yet been studied, though immortalized GnRH cells demonstrate *in vitro* circadian changes in their responsiveness to exogenous kisspeptin (Zhao and Kriegsfeld, 2009). What permits such a GnRH neuron gating mechanism remains unknown, though GnRH neurons express circadian clock genes *in vivo* (Hickok and Tischkau, 2010), suggesting these cells might possess circadian machinery to possibly provide intrinsic circadian regulation, but this has not been well studied and has not been teased out from possible incoming SCN input. Interestingly, conditional deletion of *Bmal* from just GnRH neurons of mice did not alter fertility, C.L. numbers, or the LH surge (Tonsfeldt et al., 2019), suggesting that intrinsic clocks with GnRH cells themselves may not be necessary for proper LH surges and ovulation.

## Progesterone’s role in E_2_ positive feedback and the LH surge

While E_2_ is clearly essential for positive feedback, we and others have demonstrated that progesterone (P_4_) and its receptor (PR) are also critical contributors to the LH surge mechanism. Classic studies showed that P_4_ treatment is able to amplify the magnitude, and in some cases advance the timing, of the LH surge induced by E_2_ (Everett, 1948; Krey et al., 1973; DePaolo and Barraclough, 1979; Levine and Ramirez, 1980; Lee et al., 1990b; Leite et al., 2016). Conversely, pharmacological blockade of progesterone signaling impaired the rodent LH surge and concurrent GnRH neuron activation (Lee et al., 1990b; Le et al., 1997; Chappell and Levine, 2000). In addition, like ERαKO mice, PR KO female mice cannot produce LH surges, even with proper sex steroid treatment (Chappell et al., 1997, 1999). Thus, P_4_ signaling is a required component of the LH surge process.

**FIGURE 5:**
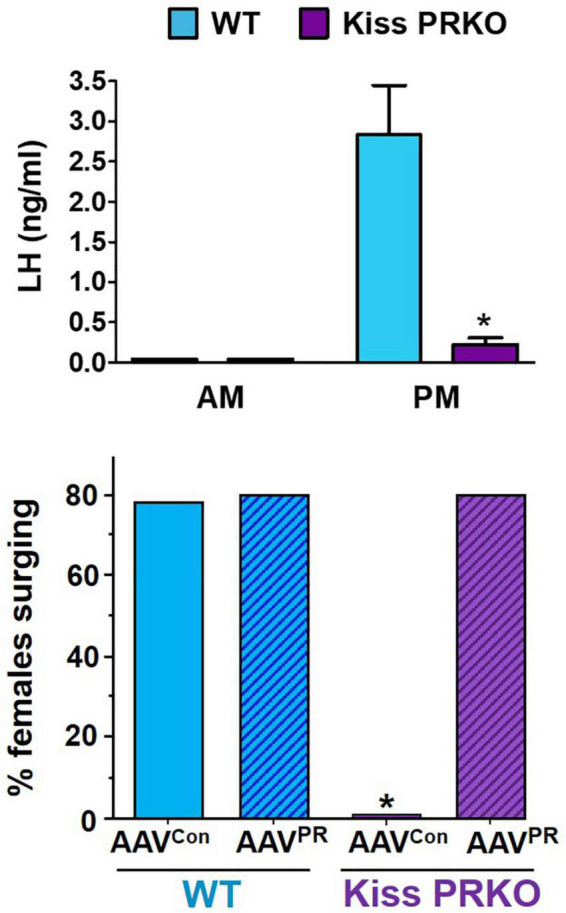
(Top) Importance of progesterone in the LH surge process as indicated by reduced PM LH surge generation in OVX + E_2_ female mice lacking PR selectively in kisspeptin cells (“Kiss PRKO”; Kiss^Cre+^/PR^flox^). Adapted from Stephens et al. (2015); Bottom. Selective re-expression of PR in just RP3V^KISS^ neurons using RP3V targeted AAVs rescues the LH surge occurrence in OVX + E_2_ Kiss PRKOs. *, Significantly different that WT controls. Adapted from Mohr et al. (2021a) AAV^con^, viral delivery of control construct. AAV^PR^, viral delivery of Cre-dependent PR construct. WT, Kiss^Cre–^/PR^flox^ females.

As with ERα, GnRH cells lack PR, indicating P_4_ acts on “upstream” brain circuitry to regulate the GnRH surge. One such candidate target area is the RP3V region, and specifically, RP3V^KISS^ neurons within it. Chappell and colleagues first tested whether the obligatory P_4_ action might occur in the general RP3V region; the authors infused PR antisense oligonucleotides into the third ventricle adjacent to the RP3V (termed AVPV in their study) to prevent PR expression in just that area. Unlike control rats, female rats infused with PR antisense oligos near the RP3V did not exhibit any LH surges, suggesting that P4 acts somewhere in that region to influence surge generation (Chappell and Levine, 2000). Because RP3V^KISS^ neurons highly express PR (Clarkson et al., 2008; Zhang et al., 2014; Stephens et al., 2015; Stephens and Kauffman, 2021), our group tested whether PR signaling specifically in kisspeptin cells is required for the LH surge (Stephens et al., 2015). We found that OVX + E_2_ transgenic mice with selective KO of PR in just kisspeptin cells (termed “KissPRKO” mice) did not show LH surges or proper RP3V^KISS^ neuron activation, as measured by *cfos* coexpression (Stephens et al., 2015; Figure 5). Along with their impaired LH surges, KissPRKO females also displayed reduced numbers of corpora lutea (an indicator of ovulation) and reduced fecundity in mating trials (Stephens et al., 2015). This finding indicated that endogenous P_4_ signaling directly in kisspeptin cells is necessary for proper E_2_-induction of the LH surge, likely by facilitating RP3V^KISS^ cell activation. A subsequent study from another group confirmed these results using a similar mouse model (Gal et al., 2016).

While the KissPRKO studies discussed above indicated that endogenous P_4_ signaling in kisspeptin cells is required for the LH surge, neither study could pinpoint the specific kisspeptin neural population that is necessary for this direct P_4_ action because PR was deleted in all kisspeptin neurons. Thus, we recently tested if selective re-introduction of PR into just one kisspeptin population of KissPRKOs would rescue the ability to generate LH surges (Mohr et al., 2021a). Given the abundant data implicating the RP3V^KISS^ population in the LH surge, we hypothesized that PR acts directly in those specific neurons to promote the surge. We therefore used targeted RP3V infusions of Cre-dependent AAVs to re-introduce the PR gene (*Pgr*) selectively in RP3V^KISS^ neurons of KissPRKO females, while leaving PR deleted from all other kisspeptin populations (Mohr et al., 2021a). This selective re-introduction of PR into just RP3V^KISS^ neurons was able to fully restore both the magnitude and prevalence of E_2_-induced LH surges (Mohr et al., 2021a; Figure 5). This exciting outcome suggests that RP3V^KISS^ neurons are indeed direct targets of P_4_ action and sufficient for PR’s positive action on the LH surge process (Figure 4).

PR KO and KissPRKO studies indicate that P_4_ is needed for proper LH surge generation. However, given that those PR KO and KissPRKO females were OVX + E_2_ with no ovarian P_4_ or treatment with exogenous P_4_, it suggests that endogenous P_4_ of non-ovarian origin is necessary for the surge. Where is such P_4_ coming from to regulate the surge? Interestingly, intriguing evidence spearheaded by the labs of Micevych and Sinchak suggests that the P_4_ involved in this process may be of neural origin. Pharmacological blockade of P_4_ synthesis or action in the brain prevents LH surges in rats (Micevych et al., 2003; Chuon et al., 2022), indicating a necessary role not just for P_4_ signaling but for P_4_ derived specifically in the brain. Indeed, insightful *in vitro* evidence from primary rodent astrocyte cultures shows that E_2_ induces *de novo* brain synthesis of P_4_ (“neuroP”) from ERα-expressing astroglia harvested from the POA region (which contains the RP3V; Sinchak et al., 2003; Micevych et al., 2007; Kuo et al., 2009, 2010a,2010b; Mohr et al., 2021b). Mirroring the *in vitro* data, *in vivo* E_2_ similarly increases P_4_ levels in the POA region of mice and rats (Mohr et al., 2019, 2021b). This “astrocrine hypothesis” posits that E_2_ increases cytoplasmic Ca^2+^ levels in RP3V astrocytes to facilitate neuroP synthesis, which then diffuses out of the glia and acts in a paracrine manner on nearby PR-expressing RP3V^KISS^ neurons (Micevych et al., 2003; Micevych and Sinchak, 2008a,^2011^; Kuo and Micevych, 2012; Mittelman-Smith et al., 2017, 2018; Mohr et al., 2019; Sinchak et al., 2020). E_2_ is proposed to exert this effect on astroglia P_4_ synthesis *via* membrane-associated ERα (Micevych and Sinchak, 2008b; Kuo et al., 2009, 2010a,2010b; Chen et al., 2014) and not ERβ or GPR30, which are also present in astrocytes. If so, it suggests that ERα plays a dual role in the positive feedback process by acting in both astrocytes and RP3V^KISS^ neurons.

## A final thought: Do non-RP3V^KISS^ neurons also participate in the LH surge?

The roles of the RP3V kisspeptin population and SCN has been frequently studied in relation to estrogen positive feedback control of LH surges in rodents. However, this does not exclude the possible involvement of other brain populations in driving or modulating the activation of GnRH neurons during the surge. Indeed, whether or not other kisspeptin neurons outside the RP3V are also involved in the LH surge is not entirely known. Although the evidence mounted in favor of a role of RP3V^KISS^ in the surge event is compelling thus far, similar evidence suggesting an important direct role of ARC^KISS^ in this process has not been abundant. ARC^KISS^ neurons are strongly implicated in governing GnRH pulses, which then stimulate downstream tonic (pulse) LH and FSH section. Because LH and FSH are both required for ovarian E_2_ synthesis and secretion, the ARC^KISS^ population is therefore *indirectly* required for preovulatory LH surges in so much as it is required for stimulating the tonic gonadotropin secretion that activates the ovaries. However, whether ARC^KISS^ neurons play a more direct role in the GnRH surge mechanism within the brain, separate form stimulating GnRH pulses, is less certain. Indeed, there is some contradictory evidence on this possibility. The three most compelling pieces of data arguing against a critical role of ARC^KISS^ in the neural GnRH surge process include are that (1) elevated E_2_ inhibits *Kiss1* (and *Tac2* and *Pdyn*) gene expression in the ARC (Smith et al., 2005a; Gottsch et al., 2009; Navarro et al., 2009), effectively reducing kisspeptin, NKB, and dynorphin synthesis under hormonal conditions when the surge occurs; (2) ARC^KISS^ neurons do not show increased *cfos* induction during the surge or between diestrus and proestrus as occurs in AVPV^KISS^ cells (Adachi et al., 2007), indicating ARC^KISS^ neuron activation is not increased during the surge event; and (3) long-term fiber photometry recording of *in vivo* ARC^KISS^ neuron activation (which correlates strongly with occurrence of LH pulses) over the course of the female mouse estrous cycle) demonstrates no increase in activation during the afternoon or evening of proestrus when the LH surge occurs (McQuillan et al., 2019). This collective evidence suggests that ARC^KISS^ neurons may not be a required player in the neural LH surge mechanism in rodents. Supporting this, ablation of the majority of ARC^KISS^ neurons in female rats does not prevent the normal occurrence of E_2_-induced LH surges (Helena et al., 2015; Mittelman-Smith et al., 2016).

Despite the evidence above that ARC^KISS^ is likely not required in the LH surge neural mechanism, several studies have proposed that ARC^KISS^ may play a modulatory role of the surge, based on limited evidence. First, ARC^KISS^ neurons are shown to project not only to GnRH dendron terminals, but also to other brain areas, including (but not limited to) RP3V^KISS^ neurons (Qiu et al., 2016). While purely anatomical, this evidence at least supports a possibility, yet to be tested, that ARC^KISS^ neurons may modulate RP3V^KISS^ neurons *via* the former’s ongoing “basal” activity and, perhaps, *via* glutamate signaling (Qiu et al., 2016); if so, enhanced activation of ARC^KISS^ neurons at the time of the surge may not be requisite for such modulatory effects, but this requires further examination. Second, female rats sustaining ablation of their ARC^KISS^ neurons show E_2_-induced LH surges of higher magnitude than females with an intact ARC^KISS^ population (Helena et al., 2015; Mittelman-Smith et al., 2016). The authors interpret this outcome to indicate there may normally be some inhibitory factor released by ARC^KISS^ neurons which serves to curb the amplitude of the LH surge, and in the absence of ARC^KISS^ neurons, this inhibition is removed, resulting in a higher surge. One of these studies proposed that dynorphin released from ARC^KISS^ neurons may be this inhibitory factor (Helena et al., 2015). However, given the reported lack of ARC^KISS^ neuron activation at the time of the surge, it is unclear how dynorphin would be secreted from those neurons to achieve this effect. Moreover, *Pdyn* levels in the ARC are strongly reduced in the presence of elevated E_2_, and ARC dynorphin levels would therefore be low at the time of surge. Finally, a recent study using optogenetics in female mice reported that experimental activation of ARC^KISS^ neurons for 2 h elicits a robust increase in serum LH that resembles an LH surge-like secretion (Lin et al., 2021). The authors proposed that ARC^KISS^ neurons may therefore amplify the LH surge. Alternatively, it is also possible that experimentally forcing ARC^KISS^ neurons to strongly fire for a sustained period of time would cause a corresponding sustained activation of GnRH neuron dendron terminals and prolonged high GnRH secretion, leading to a large secretion of LH. Such LH release may appear like an LH surge but may not represent the output of the LH surge mechanism which likely involves activation of GnRH soma rather than dendron terminals (Wang et al., 2020). Thus, while activating ARC^KISS^ neurons is sufficient to induce strong LH secretion (as expected), this does not implicate the ARC^KISS^ population in the normal endogenous GnRH surge mechanism. Moreover, if ARC^KISS^ neurons provide amplification of the surge, ablation of such neurons would be predicted to reduce the surge amplitude, but the rat studies reported an enhanced surge magnitude in the absence of ARC^KISS^ neurons (Helena et al., 2015; Mittelman-Smith et al., 2016). Finally, although ARC-specific *Kiss1* mRNA knockdown in female rats caused a lower LH surge amplitude (with normal surge incidence; Hu et al., 2015), these females were ovary-intact; thus, the 32% ARC *Kiss1* knockdown likely impacted downstream endogenous ovarian E_2_ synthesis, which may explain the observed reduction in surge magnitude.

Overall, while the current data suggesting that ARC^KISS^ neurons are not necessary for the normal surge mechanism may be more compelling than the other side of the argument, clearly more studies are needed to directly resolve this issue, and it remains possible that ARC^KISS^ neurons provide some non-requisite modulatory role. It should also be reiterated that in some other species, such as sheep and monkeys, ARC^KISS^ neurons are better implicated in the LH surge mechanism, though rostral hypothalamic kisspeptin neurons (similar to RP3V^KISS^) also show activation in these species and may also be involved (Smith, 2009; Smith et al., 2010; Watanabe et al., 2014; Vargas Trujillo et al., 2017).

Finally, we first described the presence of a small estrogen-sensitive kisspeptin population in the medial amygdala region of rodents (Kim et al., 2011). MeA^KISS^ neurons are more prevalent in males than females, but show moderately increased *Kiss1* levels in the presence of E_2_ and on proestrus versus diestrus (Kim et al., 2011; Stephens et al., 2016, 2018). Whether this small population of MeA^KISS^ neurons play a role in HPG axis regulation specifically during the LH surge remains unknown. A few studies in mice have experimentally activated MeA^KISS^ neurons *via* optogenetics or chemogenetics but reported only minor increases in LH secretion (Fergani et al., 2018; Aggarwal et al., 2019; Lass et al., 2020); notably, the pattern of LH release elicited did not resemble a large LH surge profile, suggesting that MeA^KISS^ neurons are not major players in the E_2_-induced LH surge mechanism. It remains possible MeA^KISS^ may play a modulatory role in pheromone-induced LH surges induced by conspecific exposure or in aspects of socio-sexual behavior (Adekunbi et al., 2018; Aggarwal et al., 2019), though the data thus far are very limited and more supporting evidence is needed to evaluate such possibilities.

## Author contributions

The author confirms being the sole contributor of this work and has approved it for publication.

## Conflict of interest

The author declares that the research was conducted in the absence of any commercial or financial relationships that could be construed as a potential conflict of interest.

## Publisher’s note

All claims expressed in this article are solely those of the authors and do not necessarily represent those of their affiliated organizations, or those of the publisher, the editors and the reviewers. Any product that may be evaluated in this article, or claim that may be made by its manufacturer, is not guaranteed or endorsed by the publisher.
